# Supplementary figures and images for: Trends and Changes in Socio-Economic Inequality in Self-Rated Health among Migrants and Non-Migrants: Repeated Cross-Sectional Analysis of National Survey Data in Germany, 1995–2017
Source: Int J Environ Res Public Health. 2022 Jul 7;19(14):8304. doi: 10.3390/ijerph19148304 (PMC9317826; doi:10.3390/ijerph19148304)

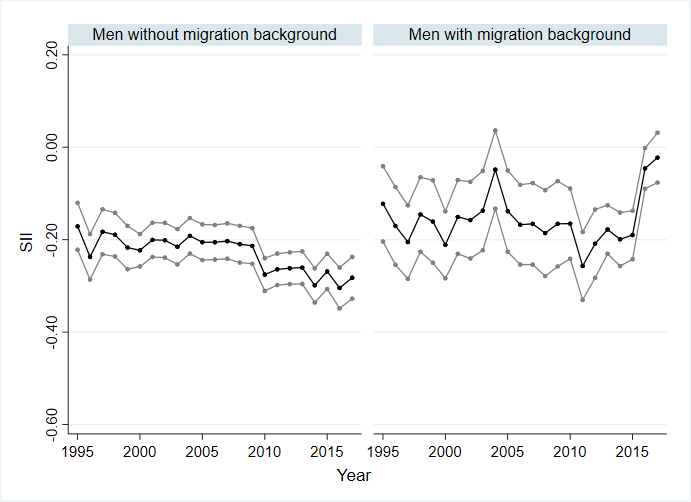

Supplement: Supplementary file 1 [file ijerph-19-08304-s001.zip › FigureS1.png]

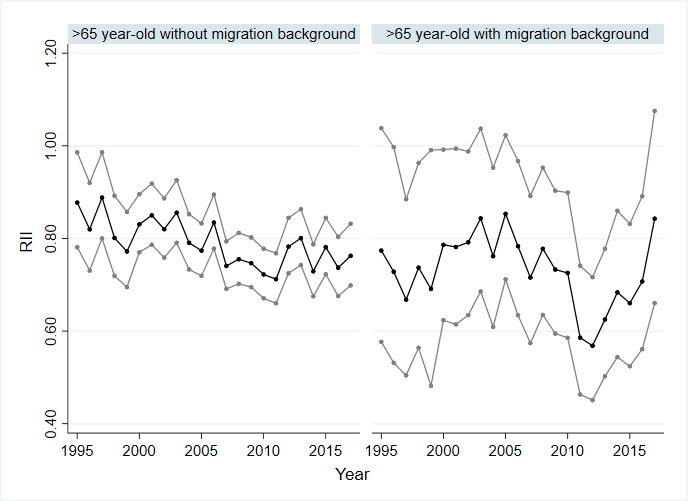

Supplement: Supplementary file 1 [file ijerph-19-08304-s001.zip › FigureS10.png]

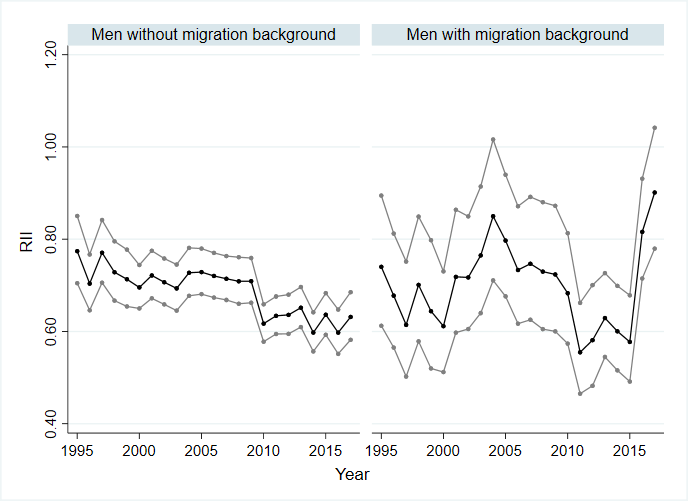

Supplement: Supplementary file 1 [file ijerph-19-08304-s001.zip › FigureS2.png]

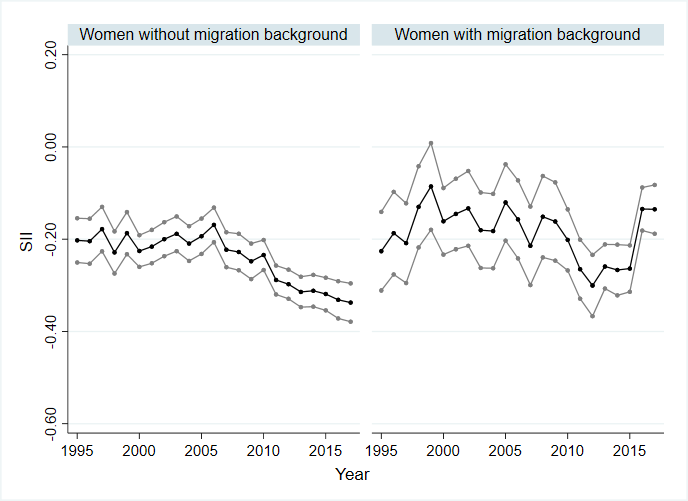

Supplement: Supplementary file 1 [file ijerph-19-08304-s001.zip › FigureS3.png]

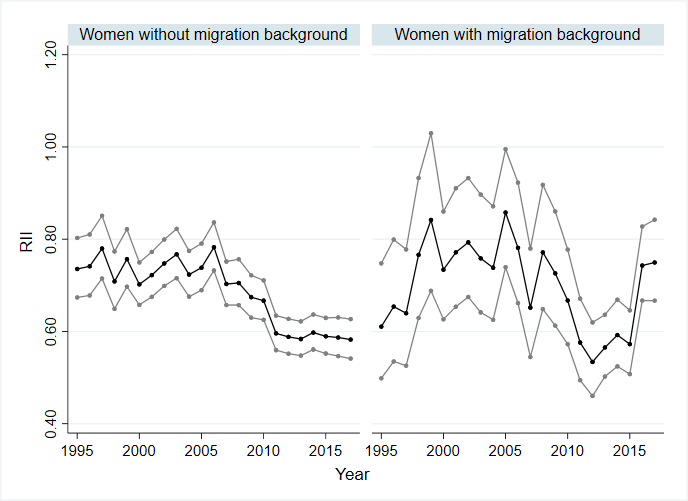

Supplement: Supplementary file 1 [file ijerph-19-08304-s001.zip › FigureS4.png]

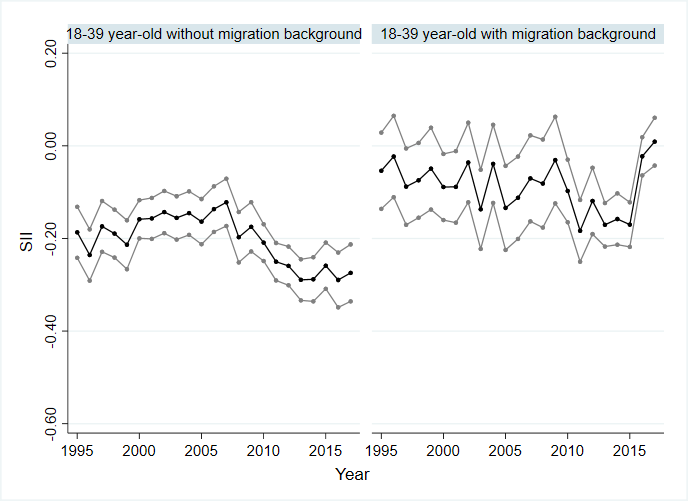

Supplement: Supplementary file 1 [file ijerph-19-08304-s001.zip › FigureS5.png]

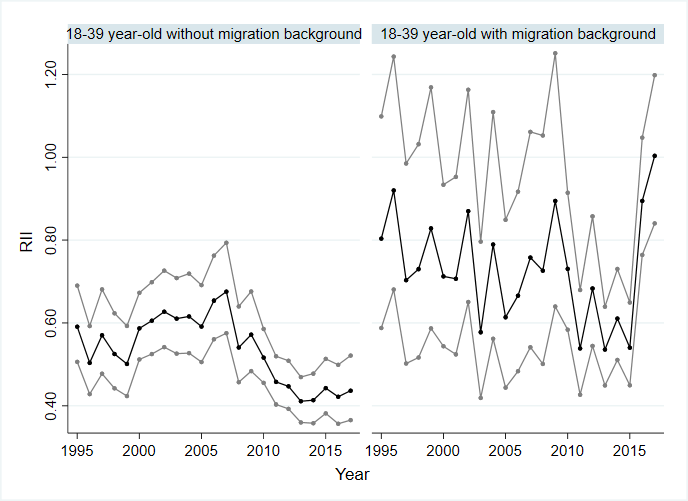

Supplement: Supplementary file 1 [file ijerph-19-08304-s001.zip › FigureS6.png]

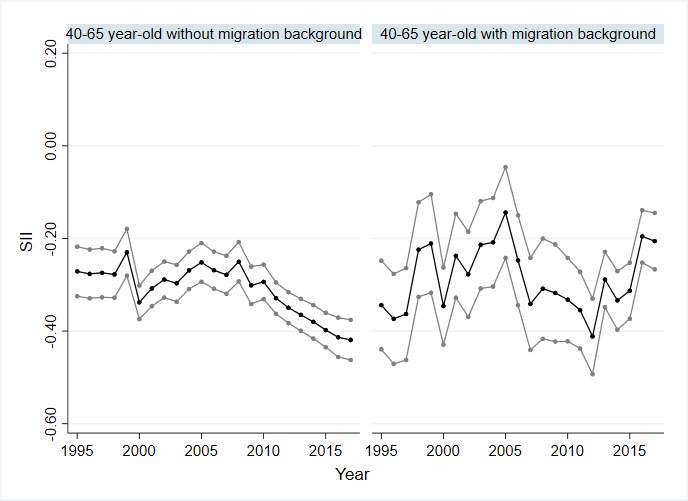

Supplement: Supplementary file 1 [file ijerph-19-08304-s001.zip › FigureS7.png]

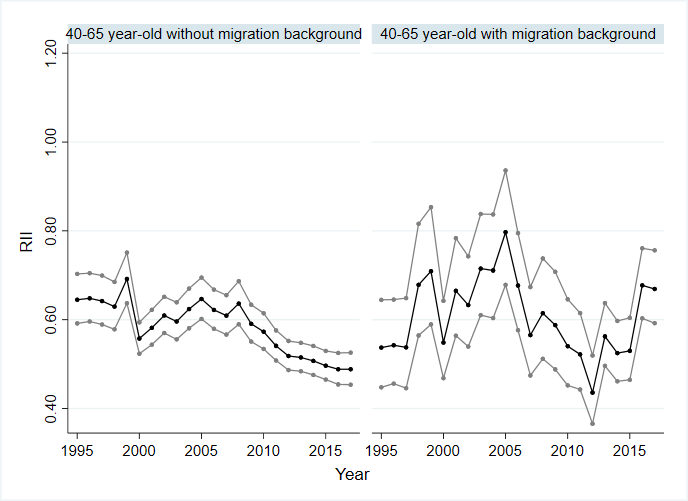

Supplement: Supplementary file 1 [file ijerph-19-08304-s001.zip › FigureS8.png]

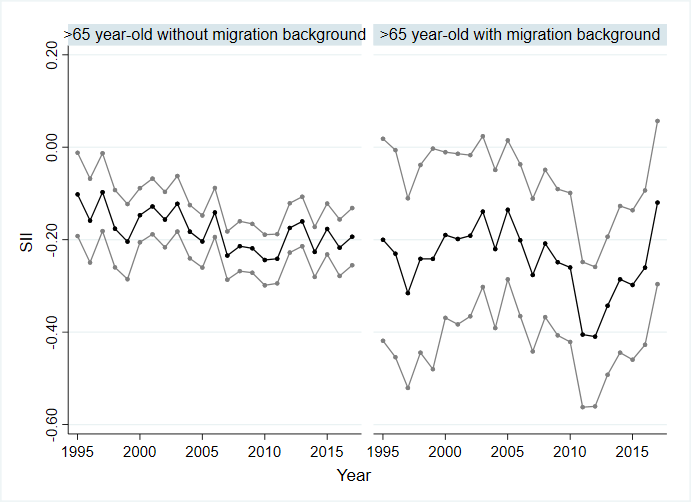

Supplement: Supplementary file 1 [file ijerph-19-08304-s001.zip › FigureS9.png]
